# Indirect ELISA Using Multi-Antigenic Dominants of 3AB and 3C Recombinant Protein to Detect Antibodies Against Senecavirus A in Pigs

**DOI:** 10.3390/vetsci12111046

**Published:** 2025-11-01

**Authors:** Dexin Li, Junhua Deng, Zenglin Wang, Yunjing Zhang, Yufang Li, Liying Hao, Zhenbang Zhu, Kegong Tian, Xiangdong Li

**Affiliations:** 1 Jiangsu Co-innovation Center for Prevention and Control of Important Animal Infectious Diseases and Zoonoses, College of Veterinary Medicine, Yangzhou University, Yangzhou 225009, China; lidexin0123@163.com (D.L.); 18763641225@163.com (Z.W.); 2 Luoyang Putai Biotechnology Co., Ltd., Luoyang 471003, China; 3 National Research Center for Veterinary Medicine, Luoyang 471003, China; 4 Joint International Research Laboratory of Agriculture and Agri-Product Safety, the Ministry of Education of China, Yangzhou University, Yangzhou 225009, China

**Keywords:** Senecavirus A, tandem antigen, indirect ELISA, serological survey

## Abstract

Senecavirus A (SVA) causes a vesicular disease in pigs with clinical signs indistinguishable from those of other swine vesicular diseases. To enable serological differentiation infected from vaccinated animals (DIVA), we developed indirect ELISAs (iELISAs) based on recombinant non-structural proteins (NSPs). A His-tagged tandem antigen, r3AB-3C, was designed by integrating immunodominant B-cell epitopes from 3AB and 3C proteins, and was successfully expressed in *Escherichia coli* (*E. coli*) and purified alongside the individual r3AB and r3C proteins. Serological evaluation results showed that the immunoreactivity of the r3AB-3C iELISA was superior to that of r3AB, which in turn was better than r3C. The r3AB-3C and r3AB iELISAs were subsequently validated. The cut-off values were established at sample-to-positive (S/P) ratios of ≥0.2635 for the r3AB-3C iELISA and ≥0.5775 for the r3AB iELISA. The r3AB-3C iELISA demonstrated higher sensitivity for detecting infection-induced antibodies than the r3AB iELISA, despite the later seroconversion of anti-NSP antibodies compared to neutralizing antibodies. In a serosurvey, the r3AB-3C iELISA revealed seropositivity rates of 35.2% in 2023 and 22.3% in 2024. In conclusion, the r3AB-3C iELISA is a valuable serological tool for monitoring SVA infection, effectively supporting DIVA strategies.

## Simple Summary

Senecavirus A (SVA) is a newly emerging virus that causes vesicular lesions in pigs, with symptoms similar to those of other vesicular diseases, making it challenging to diagnose and control. A crucial aspect of controlling SVA through vaccination is the ability to distinguish the infected pigs from the vaccinated pigs. To address this, we developed a serological tool by creating a well-designed protein, 3AB-3C, as the core component of the assay. Our findings demonstrate that the assay was highly specific and sensitive to detect infections reliably. When applied to over 3800 samples, it proved effective for large-scale serological monitoring. Overall, this assay serves as a key asset for monitoring viral spread, assisting in disease control, and bolstering vaccination programs.

## 1. Introduction

Senecavirus A (SVA), the sole member of the genus *Senecavirus* within the family *Picornaviridae*, is a non-enveloped virus with icosahedral symmetry and a diameter of approximately 27–30 nm [1]. The virus was first identified in 2002 as the SVV-001 isolate [2]. Initially investigated as an oncolytic agent for cancer therapy [1,3], SVA was later recognized as a pathogen of swine, causing vesicular lesions clinically indistinguishable from those of foot-and-mouth disease (FMD) and swine vesicular exanthema (SVE), which complicates differential diagnosis [4]. Since the first outbreak was reported in Canada in 2008 [3,5], SVA has been documented in numerous countries, including the United States [6], Brazil [7], Thailand [8], Colombia [9], and China [10], posing a significant threat to the global swine industry. Notably, recent studies have reported the detection of SVA in cattle, and the virus has been shown to infect cattle and induce vesicular clinical signs, suggesting a potential expansion of its host range [11]. The rapid spread of SVA in the world and evolving epidemiology emphasize the urgent need for reliable diagnostic tools to support effective monitoring and control measures.

The SVA genome is a single-stranded, positive-sense RNA molecule approximately 7.2 kb in length, containing a single large open reading frame (ORF) flanked by 5′ and 3′ untranslated regions (UTRs) [1]. This ORF encodes a polyprotein that is co- and post-translationally cleaved to produce mature viral proteins, forming a unique L-4-3-4 pattern: one leader protein (L), four structural proteins (VP1-VP4), and seven non-structural proteins (NSPs; 2A, 2B, 2C, 3A, 3B, 3C, 3D) [1,12]. The structural proteins VP1-VP3, exposed on the viral capsid surface, are the primary targets for neutralizing antibodies [13]. In contrast, NSPs are not incorporated into the virion but are essential for viral replication and pathogenesis [14]. Consequently, natural infection induces antibodies against both structural proteins and NSPs, whereas vaccination with inactivated vaccines primarily elicits a response against structural proteins. With the development and application of inactivated SVA vaccines [15], NSPs have emerged as ideal markers for a DIVA strategy [16]. This approach is well-established for other picornaviruses such as foot-and-mouth disease virus (FMDV). An indirect ELISAs based on the 3AB protein of FMDV successfully detect infection-induced antibodies without cross-reacting with vaccine-induced immunity, confirming 3AB as an excellent serological target [17,18]. Accordingly, the World Organisation for Animal Health (WOAH) manuals recognize 3AB and 3ABC among the most reliable indicators for serologically confirming FMDV infection [19], supporting the rationale for developing analogous NSP-based assays for SVA.

Current serological methods for SVA detection include fluorescent microsphere immunoassays (FMIA) [20], immunofluorescence assays (IFA) [21,22], immunohistochemistry assays (IHC) [23], and virus neutralization test (VNT) [22,24]. Among these, VNT is considered the gold standard due to its high specificity [24]. However, VNT is technically demanding and time-consuming, limiting its use for large-scale surveillance. For high-throughput screening, enzyme-linked immunosorbent assay (ELISA) offers significant advantages in simplicity, speed, and safety [25]. Although several studies have reported indirect ELISAs based on SVA structural proteins (VP1, VP2, VP3) [21,26] or the non-structural protein 3AB [16,25], as well as competitive ELISAs using whole-virus antigen [22,23,24], no research to date has involved an ELISA based on a fused SVA 3AB-3C protein. Given that such tandem antigens can incorporate multiple immunodominant epitopes and have been shown to enhance assay sensitivity for other viruses [27], we aimed to develop and validate a novel iELISA using a recombinant 3AB-3C tandem protein in this study to establish a more sensitive and specific tool for detecting antibodies induced by SVA infection and supporting large-scale seroepidemiological studies.

To enhance the accuracy of SVA serological detection, we designed a recombinant tandem antigen, r3AB-3C, by integrating immunodominant B-cell epitopes from the 3AB and 3C proteins. Therefore, an indirect ELISA was established using this antigen, which exhibited superior sensitivity and specificity compared to assays based on single antigens, establishing it as a reliable tool for SVA surveillance. Subsequently, this optimized method was applied in a comprehensive serological survey of pig sera (*n* = 3851) collected from 20 provinces across China between 2023 and 2024.

## 2. Materials and Methods

### 2.1. Viruses, Cells, and Serum Samples

The SVA CH-HNXC strain was provided by the National Research Center for Veterinary Medicine. Instituto Biologico-Rim Suino-2 (IBRS-2) cells were cultured under standard conditions: Dulbecco’s Modified Eagle’s Medium (DMEM; Gibco, Langley, OK, USA) supplemented with 8% fetal bovine serum (FBS; Biological Industries, Beit HaEmek, Israel), at 37 °C, and 5% CO_2_.

A panel of 200 porcine serum samples (100 VNT-positive for SVA and 100 VNT-negative) from 4- to 8-week-old Large White piglets from the National Research Center for Veterinary Medicine was used to determine the assay’s cut-off value.

The analytical specificity was assessed using standard antibody-positive sera against the following pathogens: foot-and-mouth disease virus (FMDV) serotypes A and O, porcine reproductive and respiratory syndrome virus (PRRSV), pseudorabies virus (PRV), porcine circovirus 2 (PCV2), porcine circovirus 3 (PCV3), classical swine fever virus (CSFV), and African swine fever virus (ASFV). These sera were sourced from Beijing Sino-science Gene Technology Co., Ltd. (Beijing, China) and the China Institute of Veterinary Drug Control (Beijing, China) (for ASFV). The FMDV serotype-specific reactivity was confirmed with commercial ELISA kits (Luoyang Putai Biotechnology Co., Ltd., Luoyang, China).

A serological investigation was carried out to describe the epidemiological characteristics and dynamics of SVA in China. From 2023 to 2024, clinical pig serum samples (*n* = 3851) were collected from 216 pig farms across various regions of China for SVA monitoring. The samples were divided into suckling piglets, nursing pigs, finishing pigs, gilts, sows, and boars according to their growth stages to analyze the geographical distribution and epidemic trends in detail. These sera were sourced from Luoyang Putai Biotechnology Co., Ltd.

### 2.2. Screening of Dominant Epitopes and Plasmid Construction

**Figure 1:**
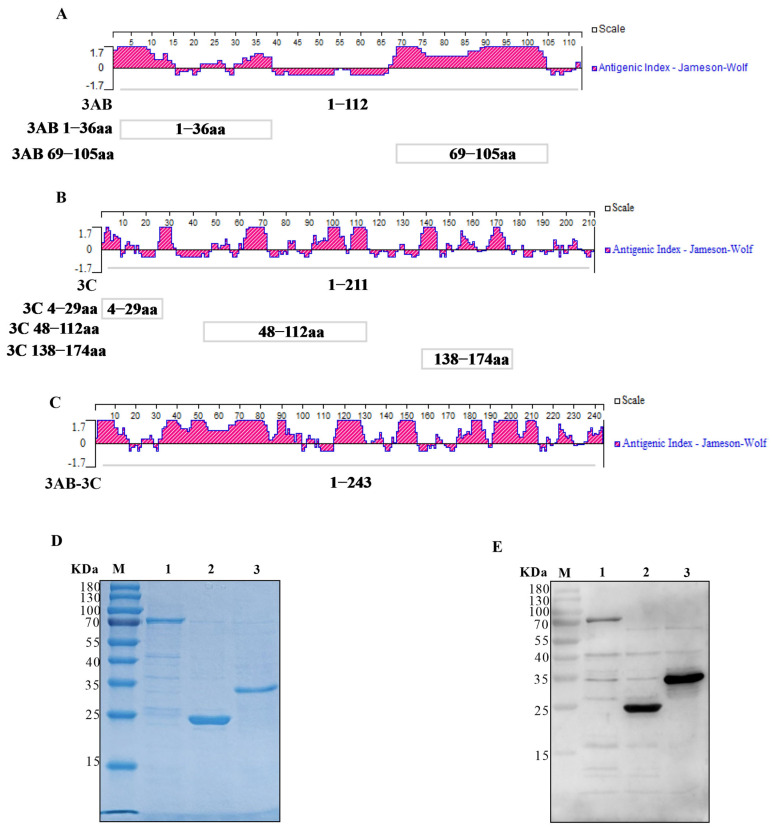
Construction, Purification, and identification of SVA tandem proteins. (**A**,**B**) Jameson-Wolf antigenicity index profiles of the 3AB (**A**) and 3C (**B**) proteins. The red regions represent the antigenicity index. Boxes indicate the analyzed epitope regions: 3AB (1–36 aa and 69–105 aa) and 3C (4–29 aa, 48–112 aa, and 138–174 aa). (**C**) Antigenic index of the designed 3AB-3C tandem protein. The antigenicity index is shown in red. (**D**) SDS-PAGE analysis of the purified recombinant proteins: 3AB (lane 1), 3C (lane 2), and 3AB-3C (lane 3). M, protein molecular weight marker. (**E**) Western blot analysis confirming the reactivity of the purified proteins (lanes 1–3, same order as in **D**) with sera from SVA-infected pigs.

The antigenic indices of the SVA 3AB and 3C proteins were predicted using both DNAStar Protean software (version 7.1.0) and the Bepipred Linear Epitope Prediction 2.0 tool (IEDB Analysis Resource). The dominant antigenic regions were selected by integrating these predictions with established epitope data from the literature [28,29]. The final selected regions (Figure 1A) for 3AB corresponded to amino acids 1–36 and 69–105, and for 3C to amino acids 4–29, 48–112, and 138–174.

For the construction of the tandem antigen, the chosen epitopes from 3AB and 3C were joined by synthetic (GGGGS)_2_ linkers to form a single recombinant gene, named the *3AB-3C* gene. This synthetic gene was codon-optimized for *E. coli*, synthesized, and subsequently cloned into the pCold-I vector by GenScript (Nanjing, China).

Additionally, the genes encoding the individual 3AB or 3C proteins were cloned into pCold-TF and pCold-I vectors, respectively. Protein expression for all constructs was performed in *E. coli* Trans BL21(DE3) plysS (TransGen Biotech, Beijing, China). All primers are listed in Table 1.

### 2.3. Expression in E. coli and Purification

Briefly, bacterial cultures were supplemented with 0.1 mg/mL ampicillin, and expression was induced with 0.2 mM isopropyl-β-D-1-thiogalactopyranoside (IPTG; Solarbio, Beijing, China) at 16 °C for 16–20 h. Following induction, the cells were harvested by centrifugation and lysed using ultrasonication. The resulting lysates were separated into a soluble supernatant and an insoluble pellet. SDS-PAGE analysis of both fractions was used to assess the solubility of the expressed proteins.

Purification of the His-tagged recombinant proteins was performed using High-Affinity Ni-NTA Resin (GenScript USA Inc., Piscataway, NJ, USA) under conditions appropriate to their solubility. For proteins found in the soluble fraction, purification was conducted under native conditions. The resin was washed with a native binding buffer (50 mM NaH_2_PO_4_, 50 mM NaCl, 10 mM imidazole, pH 8.0), and the target protein was eluted using a native elution buffer containing 250 mM imidazole. For insoluble proteins, denaturing conditions were applied. The resin-bound proteins were washed with a denaturing binding buffer (100 mM NaH_2_PO_4_, 10 mM Tris-Cl, 10 mM imidazole, 8 M urea, pH 8.0), and elution was achieved with a denaturing elution buffer containing 250 mM imidazole and 8 M urea. All procedures were conducted according to the manufacturer’s instructions.

### 2.4. SDS-PAGE and Western Blot

SDS-PAGE first separated the protein samples and subsequently transferred them to a polyvinylidene fluoride (PVDF) membrane (Millipore, Darmstadt, Germany). Thereafter, the membrane was blocked with 5% bovine serum albumin (BSA; Solarbio, Beijing, China) for 2 h at room temperature. Probing was performed by sequential incubation: first with a primary SVA-positive swine serum (1:1000 dilution in blocking buffer) for 2 h, and after washing with PBST, with an HRP-conjugated goat anti-pig IgG (H + L) secondary antibody (1:10,000; Abcam, Waltham, MA, USA) for 1 h, both at room temperature. After a final wash, the protein bands were visualized using Clarity^TM^ Western ECL Substrate (NCM Biotech, Suzhou, China) and imaged on a VILBER Fusion FX7 system (Vilber Lourmat, Collégien, France).

### 2.5. Virus Neutralization Test (VNT)

A virus neutralization test was performed using a fixed virus-diluted serum approach to determine the titers of neutralizing antibodies in porcine sera. After heat inactivation at 56 °C for 30 min, the serum samples were subjected to a two-fold serial dilution in a 96-well cell culture plate. To each dilution, an equal volume of SVA suspension (200 50% tissue culture infective dose (TCID_50_)/50 μL) was added. The mixture was incubated at 37 °C for 1 h. Following the neutralization step, an IBRS-2 cell suspension (2.5 × 10^4^ cells/well in DMEM with 8% FBS) was added to the wells. The plates were then incubated at 37 °C in a 5% CO_2_ and examined daily for cytopathic effect (CPE) for three days. The assay validity was contingent upon complete CPE in the virus control wells and the absence of CPE in the cell and serum control wells. The antibody titer was recorded as the reciprocal of the highest serum dilution that inhibited CPE in 50% or more of the cultured cells.

### 2.6. Development of iELISAs

Three iELISAs were developed for the specific detection of IgG antibodies against the recombinant SVA proteins r3AB, r3C, and r3AB-3C. Optimization of the assay parameters was performed using a checkerboard titration method with four positive and four negative serum samples, aiming for high positive-to-negative control ratios (P/N ratios) and optimal absorbance values.

Initially, the antigen coating concentration, serum dilution ratio, and secondary antibody dilution were tested under standard incubation conditions (a 30 min incubation at 37 °C each for both the serum and secondary antibody, followed by a 15 min development with 3,3′,5,5′-tetramethylbenzidine (TMB) at 37 °C). Following an initial evaluation of protein immunoreactivity, the r3AB and r3AB-3C proteins were selected for further assay refinement. The incubation times for the serum (15, 30, and 45 min) and the HRP-conjugated goat anti-pig IgG secondary antibody (15, 30, and 45 min) were then systematically evaluated. The absorbance at 450 nm was measured using a microplate reader (BioTek, Winooski, VT, USA). All commercial reagents, including buffers and ELISA plates, were supplied by Luoyang Putai Biotechnology Co., Ltd.

### 2.7. Determination of the Cut-Off Values

The cut-off values for the iELISAs based on the recombinant SVA r3AB and r3AB-3C antigens were established independently. A panel of 200 porcine serum samples was used for analysis, comprising 100 samples confirmed as positive and 100 samples confirmed as negative by the VNT.

For each serum sample, the sample-to-positive (S/P) ratio was calculated: S/P = (OD_450_ sample − OD_450_ negative control)/(OD_450_ positive control − OD_450_ negative control). Receiver operating characteristic (ROC) curve analysis was then performed on the S/P ratios for each antigen using GraphPad Prism 8.0 to assess diagnostic performance. The optimal antigen-specific cut-off value, along with its corresponding sensitivity and specificity, was determined by maximizing Youden’s index [30,31].

### 2.8. Determination of the Sensitivity, Specificity, and Repeatability

The analytical sensitivity of the iELISAs was determined by testing a two-fold serial dilution (from 1:2 to 1:128) of a known SVA antibody-positive control serum under the developed methods.

Analytical specificity was evaluated by testing the assay against antibody-positive sera for major swine pathogens, including PRRSV, PRV, PCV2, PCV3, CSFV, and ASFV. Three positive serum samples per pathogen were tested, except for FMDV serotypes O and A (*n* = 20). Further details regarding these sera are provided in Section 2.1.

Both intra-assay and inter-assay were evaluated to determine the assay’s precision. Intra-assay repeatability was assessed by testing three antisera (strong, moderate, and weak positive) eight times each within a single plate. Inter-assay reproducibility was determined by testing each of the three antisera eight times across three separate plates. For all measurements, the coefficient of variation (CV) was calculated as the standard deviation [SD/mean] × 100%.

### 2.9. Animal Experiment

Two independent animal studies were conducted to generate kinetic serum samples. The immunization study used an inactivated vaccine assessed the DIVA capability of the iELISAs, while the challenge study with live virus compared the diagnostic sensitivity of iELISAs to VNT based on seroconversion kinetics. Both the inactivated vaccine and the live virus were provided by the National Research Center for Veterinary Medicine.

Immunization Study with Inactivated Vaccine: Three 4-week-old piglets, seronegative for ASFV, FMDV, and SVA, were intramuscularly immunized with 2 mL of an inactivated SVA vaccine. The vaccine was prepared from the P9-generation SVA CH-HNXC virus (titer: 10^9.0^ TCID_50_/mL) by inactivation with 2 mmol/L binary ethylenimine (BEI; Sigma-Aldrich, St. Louis, MO, USA) at 37 °C for 36 h. The CPE was confirmed to be inactivated entirely after three blind passages on IBRS-2 cells. The antigen was emulsified with ISA 201 VG adjuvant (SEPPIC, Castres, France) before immunization. Serum samples were collected weekly via jugular venipuncture until the pigs were euthanized on 28 days post-immunization (dpi).

Challenge Study with Live Virus: Three additional SVA-seronegative pigs were intramuscularly inoculated with 2 mL per animal of the live P8-generation CH-HNXC virus (titer: 10^9.0^ TCID_50_/mL). Serum samples were collected on 0, 2, 4, 6, 8, 10, and 12 days post-challenge (dpc).

Ethical Statement: All animal experiments were performed in compliance with the ARRIVE guidelines and China’s Regulations on the Administration of Laboratory Animals. The protocol was approved by the Animal Care and Ethics Committee of the National Research Center for Veterinary Medicine (Permit No. 20170012). Procedures, including immunization and blood collection, were performed on physically restrained animals without the use of anesthesia. Licensed veterinarians carried out Euthanasia in accordance with the American Veterinary Medical Association (AVMA) Guidelines. An intravenous overdose of pentobarbital (150 mg/kg body weight; from a 200 mg/mL solution, Merck, Darmstadt, Germany) was administered via the auricular vein. Death was confirmed by the absence of pupillary light reflex and heartbeat for over 5 min and verified by two independent veterinarians.

### 2.10. Statistical Analysis

All statistical analyses and graphical generation were performed using GraphPad Prism version 8.0 (GraphPad Software, San Diego, CA, USA). Statistical differences between groups were evaluated using Student’s *t*-tests. *p*-values less than 0.05 were considered to indicate statistically significant differences: * *p* < 0.05, ** *p* < 0.01, *** *p* < 0.001; ns, not significant. All graphs were generated using GraphPad Prism.

## 3. Results

### 3.1. Epitope Analysis and Recombinant Protein Expression

Based on the identification of immunodominant B-cell epitopes (Figure 1A–C), recombinant proteins representing 3A, 3C, and a tandem 3AB-3C antigen were expressed solubly in *E. coli*. SDS-PAGE analysis showed that the purified proteins were obtained with high purity, each migrating as a single predominant band at the expected molecular weight (Figure 1D). Western blot analysis using sera from SVA-infected pigs confirmed the specific reactivity of all three recombinant proteins (Figure 1E).

### 3.2. Establishment of iELISAs and Immunoreactivity Verification of SVA Proteins

**Figure 2:**
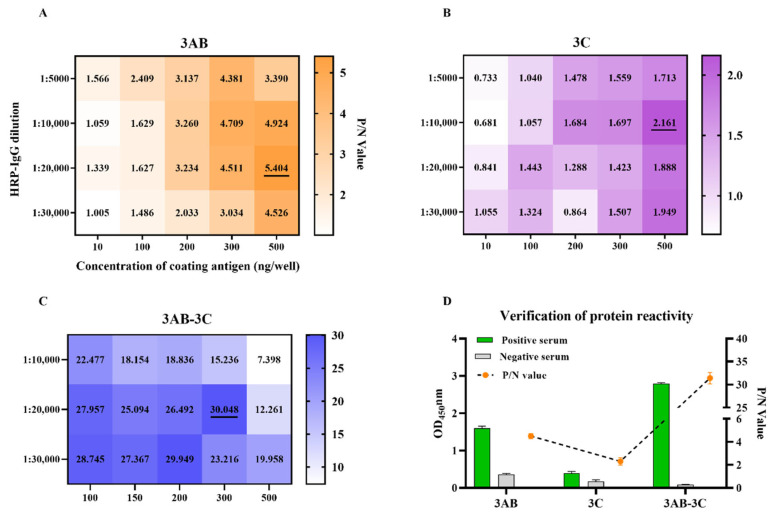
Establishment and comparative immunoreactivity of the iELISAs based on r3AB, r3C, and tandem r3AB-3C proteins. (**A**–**C**) Checkerboard titration for determining the optimal coating concentration of each antigen (r3AB, r3C, r3AB-3C) and the dilution of the secondary antibody. Heatmaps (generated with GraphPad Prism) display the corresponding positive/negative (P/N) ratios. The optimal condition is underlined in each panel. (**D**) Comparative immunoreactivity of the individual and tandem antigens. Bar graphs show the mean OD_450_ values ± SD for five SVA-positive sera (green) and three negative sera (gray). The line graph represents the corresponding P/N ratios.

The reaction conditions for the iELISAs were developed by checkerboard titration based on high P/N ratios. As shown in Figure 2A–C, the optimal coating concentrations were determined to be 500 ng/well for r3AB, 500 ng/well for r3C, and 300 ng/well for the r3AB-3C tandem protein. All assays used a uniform serum dilution of 1:50. The secondary antibody (HRP-conjugated goat anti-pig IgG) was used at dilutions of 1:20,000 for the 3AB and 3AB-3C iELISAs, and 1:10,000 for the 3C iELISA. Incubations for serum and secondary antibody were conducted at 37 °C for 30 min each, followed by a 15 min incubation with TMB substrate at 37 °C in the dark. Coating and blocking were performed at 4 °C for 24 h.

Under these conditions, the immunoreactivity of the three recombinant proteins was evaluated. The 3C protein showed consistently low P/N ratios and minimal differentiation between positive and negative sera, indicating poor diagnostic potential. In contrast, the 3AB-3C tandem protein demonstrated superior immunoreactivity compared to the 3AB protein alone, suggesting it as a more promising candidate for serodiagnosis (Figure 2D). Therefore, the 3C protein and its corresponding iELISA were excluded from subsequent analyses.

### 3.3. Assay Optimization and Cut-Off Determination for r3AB and r3AB-3C iELISAs

**Figure 3:**
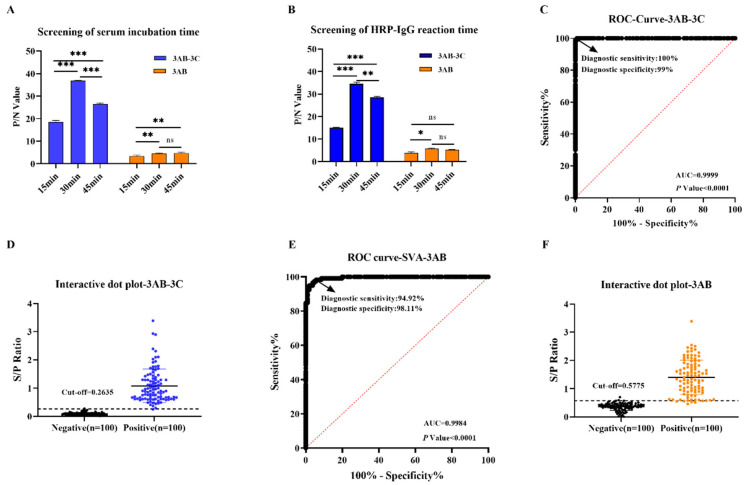
Assay optimization and determination of diagnostic cut-off values for the SVA iELISAs. (**A**,**B**) Determination of the optimal incubation times for serum (**A**) and the HRP-conjugated secondary antibody (**B**) for the iELISAs. (**C**,**D**) Diagnostic performance of the r3AB-3C-based iELISA. (**C**) Receiver operating characteristic (ROC) curve with the area under the curve (AUC). (**D**) Distribution of S/P ratios for virus neutralization test (VNT)-positive and VNT-negative serum samples. The dashed horizontal line indicates the optimal cut-off value determined by maximizing Youden’s index. (**E**,**F**) Diagnostic performance of the r3AB-based iELISA. (**E**) ROC curve. (**F**) Distribution of S/P ratios for reference serum samples. Error bars indicate the SDs of three experimental replicates. Student’s *t*-test evaluated differences, *** *p* < 0.001, ** *p * <  0.01, * *p*  <  0.05, ns, not significant.

The incubation times for serum and secondary antibody were further optimized. As shown in Figure 3A,B, 30 min was optimal for both steps in the r3AB-3C iELISA. For the r3AB iELISA, a 30 min incubation was also selected, as extending it to 45 min provided no significant improvement, favoring a shorter, standardized protocol.

The diagnostic performance of both iELISAs was evaluated using ROC curve analysis of 200 well-characterized sera (100 VNT-positive, 100 VNT-negative). The r3AB-3C iELISA achieved a near-perfect AUC of 0.9999 (95% confidence interval (CI): 0.9996–1.000; *p* < 0.0001) while the r3AB iELISA also showed excellent performance with an AUC of 0.9984 (95% CI: 0.9895–1.000; *p* < 0.0001) (Figure 3C,E). The optimal cut-off values, determined by maximizing Youden’s index, were set at S/P ≥ 0.2635 for r3AB-3C and ≥0.5775 for r3AB, yielding high sensitivity and specificity for both assays (Figure 3D,F). These results confirm the outstanding diagnostic accuracy of both tests, with the r3AB-3C iELISA exhibiting a marginally superior AUC.

### 3.4. Evaluation of the Repeatability and Reproducibility

Both iELISAs exhibited high repeatability and reproducibility, as determined by low coefficient of variation (CV) values. For the r3AB-3C iELISA, the intra-assay (repeatability) CVs ranged from 1.4% to 7.2%, and the inter-assay (reproducibility) CVs ranged from 4.1% to 8.3%. The r3AB iELISA also showed strong performance, with intra- and inter-assay CVs of 2.0–5.3% and 5.1–9.2%, respectively (Table 2). The low CV values confirm that both assays are highly precise and reliable for SVA serodiagnosis.

### 3.5. Analytical Sensitivity, Specificity, and Diagnostic Performance

**Figure 4:**
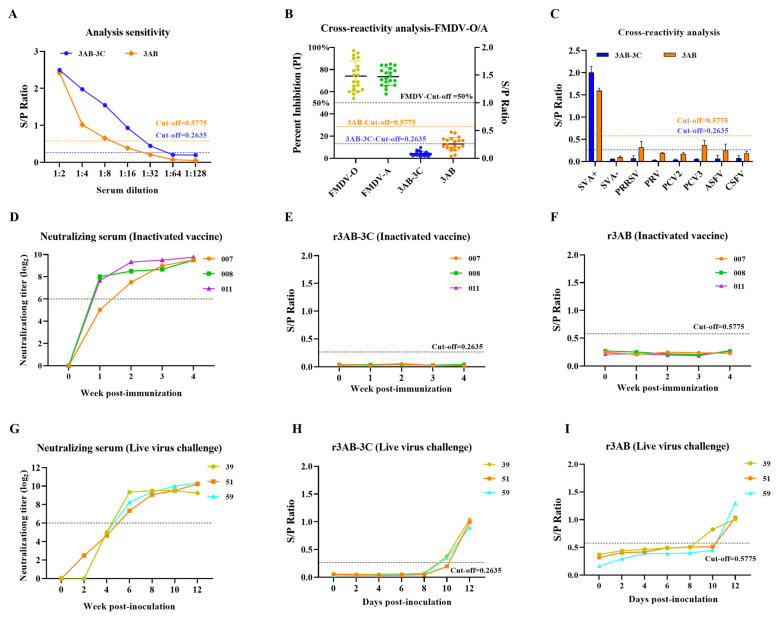
Evaluation of the analytical and diagnostic performance of the SVA iELISAs. (**A**) Analytical sensitivity was determined by two-fold serial dilution (1:2 to 1:128) of a high-titer SVA-positive serum. (**B**) Cross-reactivity assessment with FMDV serotype O/A-positive sera (*n* = 20). FMDV-specific percent inhibition (PI) values (left axis) were obtained from commercial kits, and S/P ratios for SVA antigens are shown (right axis). (**C**) Specificity evaluation using antisera against other swine pathogens (PRRSV, PRV, PCV2, PCV3, ASFV, CSFV). (**D**–**F**) Analysis of pigs immunized with inactivated SVA vaccine (*n* = 3) to confirm DIVA capability. (**D**) Virus neutralization antibody titers. (**E**,**F**) IgG antibody kinetics against (**E**) r3AB-3C and (**F**) r3AB. (**G**–**I**) Analysis of pigs from the challenge study infected with live SVA (*n* = 3) to compare diagnostic sensitivity. (**G**) Virus neutralization titers. (**H**,**I**) IgG antibody kinetics against (**H**) r3AB-3C and (**I**) r3AB. The dashed horizontal line in panels indicates the assay-specific cut-off value determined by maximizing Youden’s index.

The analytical sensitivity of the iELISAs was evaluated by testing serial dilutions of a high-titer positive serum. The r3AB-3C iELISA exhibited a four-fold higher analytical sensitivity than the r3AB iELISA, with endpoint titers of 1:32 and 1:8, respectively (Figure 4A).

Analytical specificity testing showed no cross-reactivity with sera positive for FMDV (serotypes O and A, *n* = 20) or other major swine pathogens (PRRSV, PRV, PCV2, PCV3, ASFV, CSFV) (Figure 4B,C), confirming the high specificity of both assays for SVA antibody detection.

The diagnostic performance was further assessed using sera from the vaccination and challenge studies. Sera from pigs immunized with inactivated SVA vaccine (*n* = 3) were consistently negative in both NSP-based iELISAs, confirming their ideal DIVA capability (Figure 4D–F). In pigs from the challenge study infected with live SVA (*n* = 3), antibodies against the NSPs appeared later than neutralizing antibodies. However, the r3AB-3C iELISA demonstrated superior diagnostic sensitivity by detecting seroconversion earlier (day 10 in 2/3 animals) compared to the r3AB iELISA (day 10 in 1/3 animals) (Figure 4G–I).

### 3.6. Large-Scale Serological Survey for SVA

**Figure 5:**
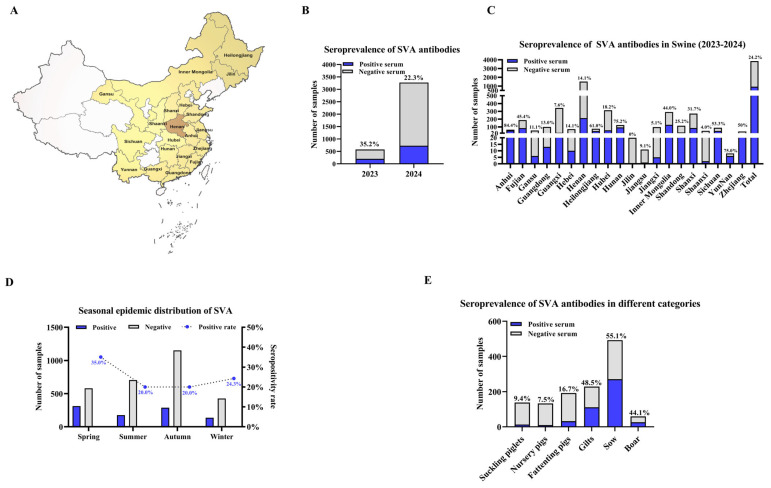
Seroepidemiological survey of SVA in Chinese swine herds (2023–2024). (**A**) Geographic distribution of sampling sites across 20 provinces in mainland China (brown shading). Sample sizes per province are: Anhui (*n* = 64), Fujian (*n* = 187), Gansu (*n* = 54), Guangdong (*n* = 100), Guangxi (*n* = 343), Hebei (*n* = 71), Henan (*n* = 1520), Heilongjiang (*n* = 77), Hubei (*n* = 314), Hunan (*n* = 125), Jilin (*n* = 20), Jiangsu (*n* = 11), Jiangxi (*n* = 98), Inner Mongolia (*n* = 291), Shandong (*n* = 115), Shanxi (*n* = 271), Shaanxi (*n* = 50), Sichuan (*n* = 90), Yunnan (*n* = 8), Zhejiang (*n* = 42). (**B**) Annual SVA seropositivity rates in 2023 and 2024. (**C**) Provincial SVA seropositivity rates. (**D**) Seasonal distribution of SVA seropositivity. (**E**) SVA seropositivity rates across different pig categories. Data are presented as the number of positive (blue) and negative (light gray) sera.

A comprehensive serological survey was conducted from 2023 to 2024 using the r3AB-3C iELISA to delineate the epidemiological status of SVA in China. A total of 3851 serum samples were obtained from 216 farms across 48 cities in 20 provinces (Figure 5A). Analysis of these samples revealed a significant decrease in seroprevalence, from 35.2% (203/577) in 2023 to 22.3% (729/3274) in 2024 (Figure 5B), resulting in an overall seropositivity rate of 24.2% (932/3851) (Figure 5C). At the farm level, the overall positive rate was 57.4% (124/216) (Table 3). Geographically, Henan Province contributed the most significant subset of samples (39.5%, 1520/3851), with a sample positivity rate of 14.1% (214/1520) (Figure 5C). Notably, 41.6% (47/102) of the farms within Henan were positive (Table 3).

The epidemic pattern exhibited an apparent seasonal variation, with higher transmission activity during winter and spring, followed by a decline in summer and autumn (Figure 5D). A marked age-associated distribution was also evident (Figure 5E). Sows exhibited the highest rate at 55.1% (271/492), followed by gilts (48.5%, 111/229) and boars (44.1%, 26/59). In contrast, much lower rates were observed in nursery pigs (7.5%, 10/133), suckling piglets (9.4%, 13/138), and fattening pigs (16.7%, 32/192).

## 4. Discussion

SVA poses a significant threat to the global swine industry, inducing clinical signs such as vesicles on the snout and coronary bands, lameness, and fever, which are indistinguishable from those caused by FMD and vesicular stomatitis (VS) [20]. This similarity complicates field diagnosis and undermines effective FMD surveillance and control programs [32]. Furthermore, with the development of various SVA vaccines, including inactivated [15], virus-like particle (VLP) [33], and epitope-based [34] vaccines, this further underscores the urgent need for reliable DIVA strategies.

NSPs, which are essential for viral replication but not packaged into virions, provide ideal markers for DIVA strategies, as they are not present in inactivated or subunit vaccines [35]. In this study, we developed an iELISA based on a recombinant tandem protein, r3AB-3C, which integrates immunodominant epitopes from the 3AB and 3C NSPs. This assay demonstrated superior sensitivity and immunoreactivity compared to iELISAs based on the single 3AB protein and 3C protein, establishing the r3AB-3C-based iELISA as a powerful tool for SVA serodiagnosis and serosurveillance.

The DIVA concept is well-established for related picornaviruses, notably FMDV, where assays based on the 3AB and 3ABC non-structural proteins are widely used [18,36]. While the 3AB protein has been explored for SVA serology [16,25], a tandem antigen combining 3AB and 3C epitopes, similar to the highly effective 3ABC of FMDV, had not been reported for SVA until now. Furthermore, evidence suggests that prokaryotically expressed SVA NSPs perform comparably to their eukaryotic counterparts in ELISA [16], supporting the feasibility of a bacterial expression system.

Based on the background, we designed a tandem antigen, r3AB-3C, which is based on integrated bioinformatic prediction and known immunodominance. The use of a bacterially expressed, epitope-focused tandem antigen provided a highly effective and practical strategy. The resulting r3AB-3C tandem protein exhibited significantly enhanced immunoreactivity compared to their individual r3AB or r3C proteins, a finding is consistent with previous reports indicating that, among the non-structural proteins, r3AB was more immunoreactive than r3C [25].

Evaluation with kinetic sera from animal studies confirmed the reliable DIVA capability of the assay, as antibodies against the r3AB-3C antigen were detected only in pigs infected with live virus, not in those receiving the inactivated vaccine, consistent with prior reports on SVA NSPs [16]. More significantly, our analysis of the antibody response during active infection revealed that seroconversion to NSPs, though delayed relative to neutralizing antibodies, was detected significantly earlier by the r3AB-3C iELISA than by the r3AB-based assay. Despite previous evaluations demonstrating high concordance with the VNT using clinical samples [16,25], the kinetics of NSP antibody seroconversion have not been thoroughly investigated. Our comparative analysis in a challenge model definitively establishes that the r3AB-3C tandem antigen provides the earliest detection of infection among existing NSP-based serological assays.

The r3AB-3C iELISA was applied to a large-scale serosurvey of 3851 clinical serum samples. It is important to note that the sample distribution was geographically uneven, with a substantial proportion (39.5%; 1520/3851 samples) originating from Henan Province. This imbalance may limit the precision of extrapolating the absolute prevalence rate to a national level. However, the assay consistently revealed robust epidemiological patterns across the diverse samples analyzed.

Our analysis revealed distinct age-associated seropositivity patterns, characterized by relatively low rates in suckling piglets and nursery pigs, compared to significantly higher rates in sows, gilts, and boars. This pattern suggests that maternal antibodies may confer temporary protection to offspring. At the same time, the high seropositivity in breeding stock highlights a significant risk for inter-farm transmission and complicates breeding management. Furthermore, the observed seasonal distribution of SVA seropositivity aligns with previously reported epidemiological patterns [25]. The consistency of these findings with established SVA epidemiology underscores the validity of our results and confirms the reliability of the r3AB-3C iELISA for field surveillance.

The diagnostic performance of the r3AB-3C iELISA was initially established using a specific serum panel. Given the evidence from Crawley and Wilkie that porcine immunoglobulin isotype production varies by individual and is influenced by immune polarization [37], it is plausible that such biological differences could impact serological outcomes. Therefore, extending validation to include a wider variety of swine, representing different ages, breeds, and immune backgrounds, is crucial to verify that the cut-off value remains consistent and effective across the general population.

## 5. Conclusions

In summary, we have developed an r3AB-3C iELISA as a reliable serological tool that enables the specific detection of SVA infection, thereby fulfilling the need for a DIVA-compliant assay. Despite a later seroconversion compared to neutralizing antibodies, this assay demonstrated superior sensitivity compared to a single-protein (r3AB)-based iELISA. Its successful application in a serosurvey not only characterized the current epidemiological status of SVA in China but also validated its practical utility for surveillance. The r3AB-3C iELISA will be a powerful tool in the SVA surveillance program.

## Figures and Tables

**Table 1:** Primers used for constructs.

Constructs	Primer (5′–3′)	Vector
pCold-I/pCold-TF	F: GAGCTCGGTACCCTCGAGGG	
	R: CATATGCCTACCTTCGATATGATGA	
3AB	F: ^1^ atatcgaaggtaggcatatgATGAGCCCTAATGAGAACGACGACA	pCold-TF
	R: ccctcgagggtaccgagctcTTATTGCATTTCCATAAGAGAGAGC	
3C	F: atatcgaaggtaggcatatgATGCAGCCCAACGTGGACATGGGCT	pCold-I
	R: ccctcgagggtaccgagctcTTATTGCATTGTAGTCAGAGGCTCA	
3AB-3C	^2^ /	pCold-I
	/	

Note: ^1^ Lowercase letters represent the homologous arms for vector recombination; ^2^ The symbol “/” indicates that no primer was required.

**Table 2:** Repeatability (intra-assay) and reproducibility (inter-assay) of the developed iELISAs.

SVA Antisera	Intra-Assay						Inter-Assay					
	3AB-3C			3AB			3AB-3C			3AB		
	Mean	SD	CV	Mean	SD	CV	Mean	SD	CV	Mean	SD	CV
Strongly positive	2.476	0.034	1.4%	2.101	0.042	2.0%	2.355	0.182	7.7%	2.196	0.112	5.1%
Moderately positive	0.915	0.042	4.6%	1.226	0.049	4.0%	0.911	0.038	4.1%	1.135	0.104	9.2%
Weakly positive	0.513	0.037	7.2%	0.844	0.045	5.3%	0.496	0.041	8.3%	0.885	0.062	7.0%

**Table 3:** Serologic analysis of SVA in swine farms.

Province	^a^ Positive	^a^ Negative	^a^ Total	Ratio (%)
Anhui	1	0	1	100.0%
Fujian	4	3	7	57.1%
Gansu	1	1	2	50.0%
Guangdong	1	2	3	33.3%
Guangxi	3	1	4	75.0%
Hebei	3	1	4	75.0%
Henan	47	55	102	46.1%
Heilongjiang	1	1	2	50.0%
Hubei	3	1	4	75.0%
Hunan	2	0	2	100.0%
Jilin	0	1	1	0.0%
Jiangsu	1	0	1	100.0%
Jiangxi	3	1	4	75.0%
Inner Mongolia	32	17	49	65.3%
Shandong	3	4	7	42.9%
Shanxi	12	3	15	80.0%
Shaanxi	1	0	1	100.0%
Sichuan	4	1	5	80.0%
Yunnan	1	0	1	100.0%
Zhejiang	1	0	1	100.0%
Total	124	92	216	57.4%

Note: ^a^ means the number of pig farms.

## Data Availability

The original contributions presented in this study are included in this article. Further inquiries can be directed to the corresponding authors.

## Abbreviations

The following abbreviations are used in this manuscript:

iELISA	indirect enzyme-linked immunosorbent assay
NSPs	non-structural proteins
S/P	sample-to-positive
DIVA	Differentiating Infected from Vaccinated Animals
*E. coli*	*Escherichia coli*
ORF	open reading frame
UTR	untranslated region
WOAH	World Organisation for Animal Health
IBRS-2	Instituto Biologico-Rim Suino-2
PVDF	polyvinylidene fluoride
BSA	bovine serum albumin
CPE	cytopathic effect
TCID_50_	50% tissue culture infective dose
FMIA	fluorescent microsphere immunoassays
IFA	immunofluorescence assay
IHC	immunohistochemistry assay
VNT	virus neutralization test
SVA	Senecavirus A
FMDV	foot-and-mouth disease virus
SVE	swine vesicular exanthema
PRRSV	porcine reproductive and respiratory syndrome virus
PRV	pseudorabies virus
PCV2	porcine circovirus 2
PCV3	porcine circovirus 3
CSFV	classical swine fever virus
ASFV	African swine fever virus
IPTG	isopropyl-β-D-1-thiogalactopyranoside
P/N	positive-to-negative control
dpi	days post-immunization
dpc	days post-challenge
